# Microbiota and Extracellular Vesicles in Anti-PD-1/PD-L1 Therapy

**DOI:** 10.3390/cancers14205121

**Published:** 2022-10-19

**Authors:** Surbhi Mishra, Sajeen Bahadur Amatya, Sonja Salmi, Vesa Koivukangas, Peeter Karihtala, Justus Reunanen

**Affiliations:** 1 Biocenter Oulu & Cancer and Translational Medicine Research Unit, University of Oulu, 90014 Oulu, Finland; 2 Department of Surgery, Oulu University Hospital, University of Oulu, 90014 Oulu, Finland; 3 Medical Research Center Oulu, Oulu University Hospital, University of Oulu, 90014 Oulu, Finland; 4 Helsinki University Hospital Comprehensive Cancer Center, University of Helsinki, 00029 Helsinki, Finland

**Keywords:** extracellular vesicles, microbiota, anti-PD therapy, cancer

## Abstract

**Simple Summary:**

Immune checkpoint inhibitors (ICI) targeting PD-1/PD-L1 have emerged as contemporary treatments for a variety of cancers. However, the efficacy of antibody-based ICIs could be further enhanced. Microbiota have been demonstrated to be among the vital factors governing cancer progression and response to therapy in patients. Bacteria secrete extracellular vesicles carrying bioactive metabolites within their cargo that can cross physiological barriers, selectively accumulate near tumor cells, and alter the tumor microenvironment. Extracellular vesicles, particularly those derived from bacteria, could thus be of promising assistance in refining the treatment outcomes for anti-PD-1/PD-L1 therapy. The potentiality of microbiota-derived extracellular vesicles in improving the currently used treatments and presenting new therapeutic avenues for cancer has been featured in this review.

**Abstract:**

Cancer is a deadly disease worldwide. In light of the requisite of convincing therapeutic methods for cancer, immune checkpoint inhibition methods such as anti-PD-1/PD-L1 therapy appear promising. Human microbiota have been exhibited to regulate susceptibility to cancer as well as the response to anti-PD-1/PD-L1 therapy. However, the probable contribution of bacterial extracellular vesicles (bEVs) in cancer pathophysiology and treatment has not been investigated much. bEVs illustrate the ability to cross physiological barriers, assemble around the tumor cells, and likely modify the tumor microenvironment (EVs). This systematic review emphasizes the correlation between cancer-associated extracellular vesicles, particularly bEVs and the efficacy of anti-PD-1/PD-L1 therapy. The clinical and pharmacological prospective of bEVs in revamping the contemporary treatments for cancer has been further discussed.

## 1. Introduction

Cancer is a fatal disease responsible for numerous global deaths every year. According to the World Health Organization’s recent census, cancer is the root of fatality in individuals under 70 years of age in 112 countries [1]. Standard cancer treatments such as surgery, radiotherapy, and chemotherapy have been efficient in treating and alleviating most symptoms; however, they remain ineffective for nearly half of the cancer cases [2]. There is a dire need for alternative potent therapeutic methods to evade the disease entirely [3].

Targeted immunotherapies utilizing immune checkpoint blockade (ICB) have provided new therapeutic prospects for cancer patients. ICBs target immune checkpoint molecules and hinder their function. Programmed cell death protein 1 (PD-1) is one such immune checkpoint molecule. It is a receptor expressed on T cells and has a predominant ligand: programmed cell-death ligand 1 (PD-L1) [4], which is upregulated on the tumor cell surface [5]. The PD-L1 represented on tumor cells binds to its receptor PD-1 on T cells, rendering T cells unable to attack the T cells and consequently assisting the progression of tumor. In this way, PD-1 pathway regulates immune resistance within the tumor microenvironment, and its blockade can be implemented for augmenting an anti-tumor response [6].

The complexity and behavior of gut microbiota makes them analogous to a metabolic organ regulating different pathways of the whole metabolism [7]. As one acquires microbiome during development, the immune system also matures. Different members of the microbial community interact with specific immune components and affect the synthesis of anti-inflammatory and immune-regulatory cytokines. On that account, the microbiome has been demonstrated to play a vital role in cancer susceptibility and response to cancer therapies such as immune checkpoint inhibitors [8,9,10].

The mechanisms by which microbiota regulate carcinogenesis and tumor progression remain unclear to date. It is also uncertain whether the crosstalk between the intestinal or intra-tumoral bacteria and the host cells in the tumor microenvironment (TME), regulating the progression or inhibition of tumor, is mediated via secreted microbial metabolites or microbial extracellular vesicles. Microbial dysbiosis has been discovered to be a crucial factor affecting oncogenesis, advancement of the tumor, and response to therapy in many cancer types such as colorectal cancer, liver cancer, etc. [11,12,13]. Microbial dysbiosis in cancer could provoke the release of microbiota-derived bacterial extracellular vesicles (bEVs) into the circulatory system. The systematically released bEVs could then trigger tolerogenic immune reprogramming of the TME, thereby acting as tumor-promoting entities, and could also traverse to distant tissues and organs [14]. In contrast, bEVs derived from certain gram-negative bacteria such as *Vibrio cholera*, *Escherichia coli* (BL21), and *Shigella flexneri* have been found to significantly inhibit tumor growth when injected into colon tumor-bearing mice [15]. Consequently, bEVs present a promising prospect for further comprehending carcinogenesis and disease progression, improving existing immunotherapies, and formulating new targeted therapies. This review discusses the existing information on the effects of cancer-associated microbiota and bEVs in cancer etiology, tumor progression, and the response to immune checkpoint blockade therapy, and also highlights the gaps in the comprehension of bEVs as appealing diagnostic and therapeutic tools. 

## 2. Methods

**Figure 1:**
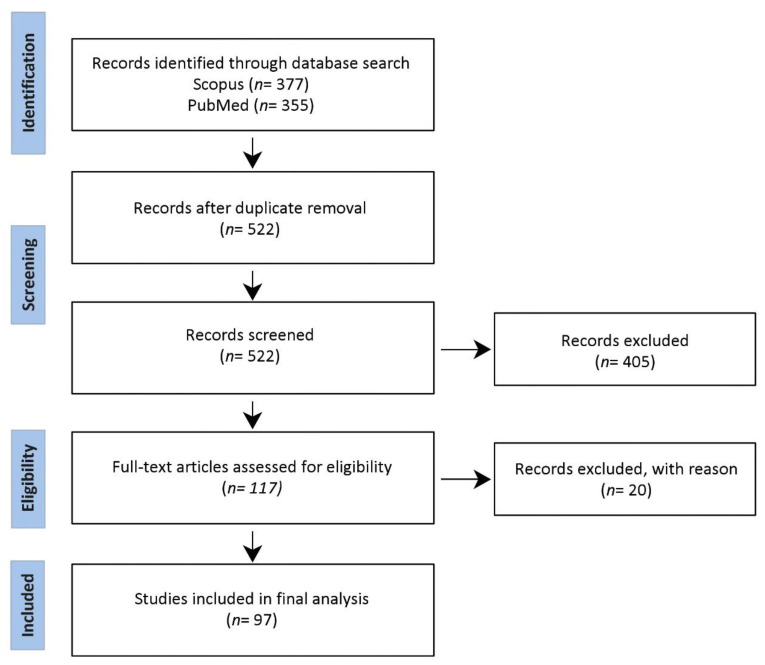
Summary of methodology.

PubMed and Scopus searches were conducted. The query combined separate search items (i) “bacteria”, (ii) “extracellular vesicle”, and (iii) “Anti-PD”. No time interval was introduced. Original publications in the English language comprised a mandate for the search. Data derived from the search was compiled into Covidence for further screening. Two independent reviewers reviewed all the candidate studies. A critical assessment of all the studies was done to filter articles relevant to the topic. Studies that explored the role of human microbiota in anti-PD-1/PDL-1 therapy and studies involving the contribution of extracellular vesicles in anti-PD-1/PDL-1 therapy responses were included. Studies related to probiotics and alternative medicines were excluded. Additional information on the methodology can be found in the Supplementary Table S1. The methodology is summarized as a flowchart in Figure 1.

## 3. Microbiota in Cancer and Anti-PD-1/PD-L-1 Therapy

### 3.1. PD-1/PD-L-1 Axis and Microbiota

The use of microbiota driven immune system activation that slows tumor progression and promotes tumor evasion has become a promising approach for cancer therapy in recent years [16]. PD-1 is an inhibitor of immune responses and is expressed by different types of immune cells [17]. PD-L1 is generally expressed by activated B cells, T cells, and macrophages. Additionally, PD-L1 is also highly expressed by tumor cells to escape anti-tumor responses. PD-L1 expressed in tumor cells binds to its receptor PD-1 in T cells, attenuating the ability of the T cells to target tumor cells, facilitating further tumor progression [18]. 

The PD-1/PDL-1 axis has been known to play a key role in the regulation of the immune system in a cancer microenvironment. Gut microbiota have been found to influence the outcomes of anti-PD-1/PD-L1 therapy, possibly by shaping host immune responses [16]. Sivan et al. (2015) compared the growth of a melanoma tumor implanted in mice derived from two different facilities: Taconic Farms (TAC) and Jackson Laboratory (JAX). The mice selected were genetically identical C57BL/6 mice, but harbored different microorganisms in the intestine. It was observed that the tumor had grown more aggressively in TAC mice than in JAX mice, and the immune cell accumulation and responses were significantly higher in JAX mice. There was a positive association between the *Bifidobacterium* species and the relative abundance of immune cells within the tumor microenvironment. When *Bifidobacterium* containing fecal material from JAX mice was fed to TAC mice, the anti-tumor immunity obtained was comparable to the effect observed during anti-PDL1 therapy. It was observed that the administration of *Bifidobacterium* in mice receiving anti-PD therapy markedly enhanced the overall response to anti-PD therapy. The authors suggested that the composition of the gut microbiome may be one of the factors influencing the spontaneous anti-tumor immunity, as well as the enhanced therapeutic effects of drugs targeting the PD-1/PD-L1 axis [19]. 

### 3.2. Microbiota in Anti-PD-1/PD-L1 Therapy

Several studies have reported that the diversity of gut microbiome and the presence of favorable commensal bacteria enhance the clinical outcomes of immune checkpoint inhibitors (ICI) [12,20,21,22]. Some bacteria are explicitly associated with patients who respond to the treatment (responders), rather than those who do not respond to the treatment (non-responders). Studies suggesting a link between human gut microbiota and clinical responses to anti–PD-1/PD-L1 treatment in diseases such as melanoma, non-small cell lung cancer (NSCLC), renal cell carcinoma (RCC), and urothelial cancers have gained recognition in the recent years [13,23] (Table 1). The microbiota in gastrointestinal tract help to maintain the intestinal mucosal barrier and intestinal homeostasis, and they also assist in anti-cancer immune surveillance in healthy individuals, through the combination of tumor antigenicity and adjuvanticity [24]. The whole or part of gut microbiota, or some of its product, may mimic tumor antigens and train immune cells against the tumor, before they enter the lymphatic system [25]. These antigens may also trigger the host systemic immune responses through pattern recognition receptors (PRR), resulting in the activation of host responses against tumor cells [26].

Gopalakrishnan et al. (2018), in their study, indicated that the responses to anti PD-1 immunotherapy may be modulated by intestinal microorganisms. They proposed that the abundance of favorable gut bacteria, such as members of the families *Ruminococcaceae* and *Faecalibacterium*, facilitates enhanced anti-tumor and systemic immune responses, resulting in increased antigen presenting capacity and enhanced effector T cell function in the tumor microenvironment and its periphery. In contrast, a relatively higher abundance of unfavorable gut bacteria such as *Bacteroidales* results in impaired anti-tumor and systemic immune responses [22]. These findings outline the significance of microbiota in immune checkpoint therapy.

The presence of a specific group of gut microorganisms has been linked with a better response to the treatment, while the treatment of patients with antibiotics prior to or during anti-PD-1 or anti-PD-L1 immunotherapy has been linked to poor clinical outcomes [30,31,32]. Patients who had not been treated with antibiotics, or who had only been treated for a short term, showed an overall longer survival than those with longer antibiotic exposures [33]. Antibiotics administered 60 days prior to the start of the immune checkpoint blockade treatment did not show a strong clinical effect, compared to those patients who had been treated with antibiotics within 30 days prior to treatment [34]. The result might be attributed to a decrease in the richness and variety of gut microorganisms during the antibiotic treatment [35,36]. 

**Figure 2:**
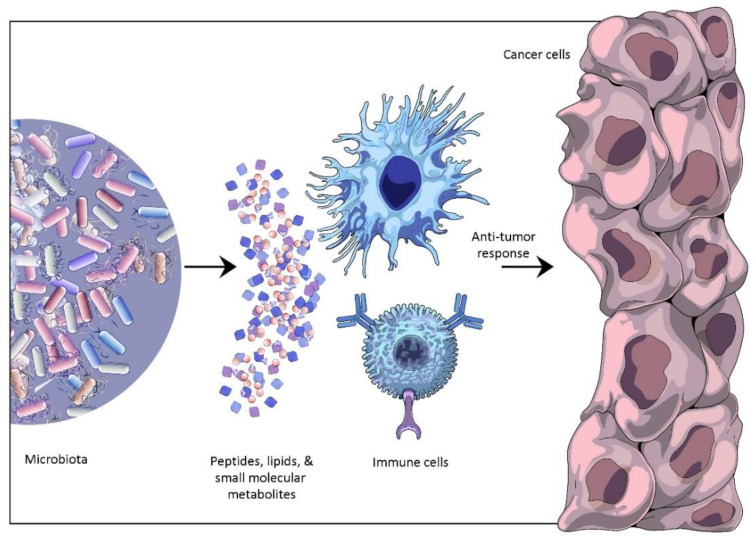
Host–microbiota interaction and anti-tumor response.

The effect antibiotics pose on the outcome of anti-PD-1/PD-L1 immunotherapy has been investigated in the case of NSCLC, RCC, and bladder cancer. Cancer patients using antibiotics 2 months before or 1 month after the start of immunotherapy showed decreased survival rates. These outcomes were attributed to gut dysbiosis resulting from the usage of antibiotics. The quantitative metagenomic analyses of the gut microorganisms before and during the immunotherapy revealed that some bacterial species, such as *Akkermansia muciniphila* and *Ruminococcus* spp., had significantly increased in the responders, in comparison to those who did not respond to the immunotherapy [13,37]. The precise mode of interaction between these microbes and the host immune system is yet to be illuminated. Possible mechanisms have been hypothesized as [38]:

(i) through the activation of immune cell responses due to presence of microbial antigens: bacterial antigens such as peptide or lipid structures can activate a large range of T cell receptors. These microbial antigens either help tumor-specific immune responses and facilitate anti-tumor activity, or in some cases may cross-react against tumor-specific antigens and in turn become responsible for anti-tumor drug resistance [25,39].
(ii) through the involvement of pattern recognition receptors: immune cells when exposed to microbes such as *Bacteroides fragilis* or *Akkermansia muciniphila* activate systemic interleukin dependent immune responses, which facilitate tumor control [13,40]. Zitvogel et al., (2018) suggested that ligands of toll-like receptors or Nod-like receptors may cause these microorganisms to produce such immune responses [38].
(iii) through small molecular metabolites produced by microbes, which may mediate a host’s systemic immune responses [25,38] (Figure 2).

One of the studies performed in NSCLC patients receiving nivolumab exhibited no change in the response rate, regardless of whether they were treated with antibiotics or not [37]. Nevertheless, patients receiving antibiotic treatment are generally in a compromised health status, which may impact the overall treatment outcome [32]. The composition of microbiota identified in a study may be influenced by the way the study was designed, such as the sample collection protocol, inclusion and exclusion criteria, the treatment regimen followed, the differences in population selected, and their diet. These variables might alter the results obtained during the study. Therefore, whether the diversity of the gut microbiota found prior to the anti-PD-1/PD-L1 therapy depicts the overall health status of the patient, or the microbial species are actually harbored to assist the immune system of the host to promote a positive response, is yet to be established [25].

The diversity and composition of microbiota differ starkly between the responders and non-responders of anti-PD-1 immunotherapy. Different studies have reported that the immune checkpoint inhibitors modify host microbiota composition, which in turn influences the treatment outcomes. Peng et al. (2020) reported that patients with gut microbiota in gastrointestinal cancer receiving checkpoint inhibitors had significantly higher abundance of *Prevotella*, *Ruminococcaceae* and *Lachnospiraceae* in their gut [25]. The abundance of specific gut bacterial species such as *Bacteroides thetaiotaomicron*, *Bacteroides fragilis*, and *Burkholderia cepacia* were also found to be increased in presence of ICI ipilimumab in some animal models [40]. These gut microbial alterations presumably improve the anticancer effect of the ICIs.

### 3.3. Effect of Fecal Microbiota Transplant (FMT) on Anti-PD-1 Therapy

Fecal microbiota transplantation (FMT) of gut commensal microorganisms from patients that responded to immune therapy has been known to promote anti–PD-1 efficacy [12,13] (Table 2).

**Figure 3:**
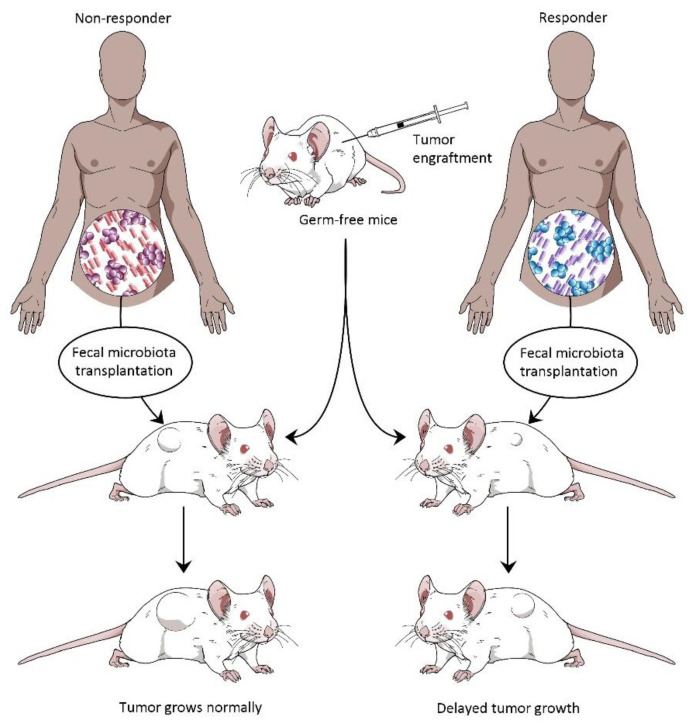
Effect of fecal microbiota transplantation (FMT) from responders and non-responders of anti-PD-1 therapy in tumor-engrafted germ-free mice.

Gopalakrishnan et al. (2018) in their study reported that when germ-free mice were transplanted with fecal material from human patients that responded to anti–PD-1 therapy, they showed improved responses to anti–PD-L1 therapy. The study also reported significant reduction in tumor size compared to mice transplanted with fecal material from patients that did not respond to anti–PD-1 therapy. Matson et al. (2018) reported that fecal material from metastatic melanoma patients who responded to anti-PD-1 treatment when transplanted to germ-free mice slowed the tumor growth. Due to the transfer of beneficial bacteria that influence anti-tumor immunity from the patients, the fecal material was able to induce a partial anti-tumor effect in the mice [12]. Routy et al. (2018) reported that fecal material from NSCLC patients who responded well to anti-PD-1 treatment when transplanted to germ-free mice resulted in delayed tumor growth, as compared to the mice fed with fecal material from patients who did not respond to the anti-PD-1 therapy [13] (Figure 3).

Only a few FMT investigations involving human clinical trial have been performed to date. Baruch et al. (2021) performed a study to access how safe and feasible the FMT treatment is when it is combined with anti-PD-1 immunotherapy. Out of ten patients with anti-PD-1-refractory metastatic melanoma, one patient showed a complete response while two patients showed partial responses. The individuals who had previously been complete responders of anti-PD-1 monotherapy for a year were selected as FMT donors. The FMT treatment was associated with favorable changes in gene expression profiles and immune cell infiltration in both the tumor microenvironment and gut lamina propria [29]. Davar et al. (2021) administered a combination of fecal material derived from responders and anti-PD-1 therapy in patients with PD-1 refractory melanoma. The combination provided clinical benefits to 6 out of 15 patients. They found that the microorganisms were able to successfully colonize the gastrointestinal tract, modified the tumor microenvironment, and overcame the anti-PD-1 drug resistance. Further investigation highlighted that the gut microbiota composition had shifted to the microorganisms such as *Firmicutes* (*Ruminococcaceae* and *Lachnospiraceae families*) and Actinobacteria (*Coriobacteriaceae* and *Bifidobacteriaceae* families), which have been previously associated with effective clinical responses to anti–PD-1 therapy [42]. Multiple cytokines and chemokines associated with anti-PD-1 resistance downregulated, while those associated with favorable clinical outcome were found to be upregulated. Failure in response to FMT treatment may be due to various reasons including the inability to replace the host microbiota and successfully implant beneficial microbiota favoring anti-PD-1 treatment into the recipient, the inability to respond to the tumor progression regardless of beneficial microbiota obtained because of the patient’s own immunodeficient status or lack of tumor immunogenicity, or a total absence of microbiota needed for anti–PD-1 therapy effectiveness in the FMT provided to them [42].

## 4. Extracellular Vesicles in Cancer and Anti-PD-1/PD-L1 Therapy

Immune checkpoint blockade therapy has evolved as a competent therapeutic aid for cancer [43,44]. Numerous factors govern the therapy response in patients, including TME, tumor mutational burden, systemic conditions, etc., while extracellular vesicles (EVs) have surfaced as the key regulators of the anti-PD-1/PD-L1 therapy response. EVs are biologically active lipid-bilayer nanovesicles secreted by various cells, including normal and tumor cells through endosomal pathways into the extracellular space [45,46,47,48,49]. They circulate in the body and transport their cargo—which is composed of DNA, RNA, proteins, small molecular metabolites, and lipids—to the target cells mediating intercellular communication. EVs are distinctly heterogeneous, and each cell type secretes a unique assortment of EV subpopulations that vary in size, content, and function. EVs are known to play a significant role in cancer initiation, metastasis, and immunity, and are explored as biomarkers for tumor diagnosis and for the assessment of responses to therapy [50,51].

### 4.1. Mammalian Extracellular Vesicles (MEVs)

**Figure 4:**
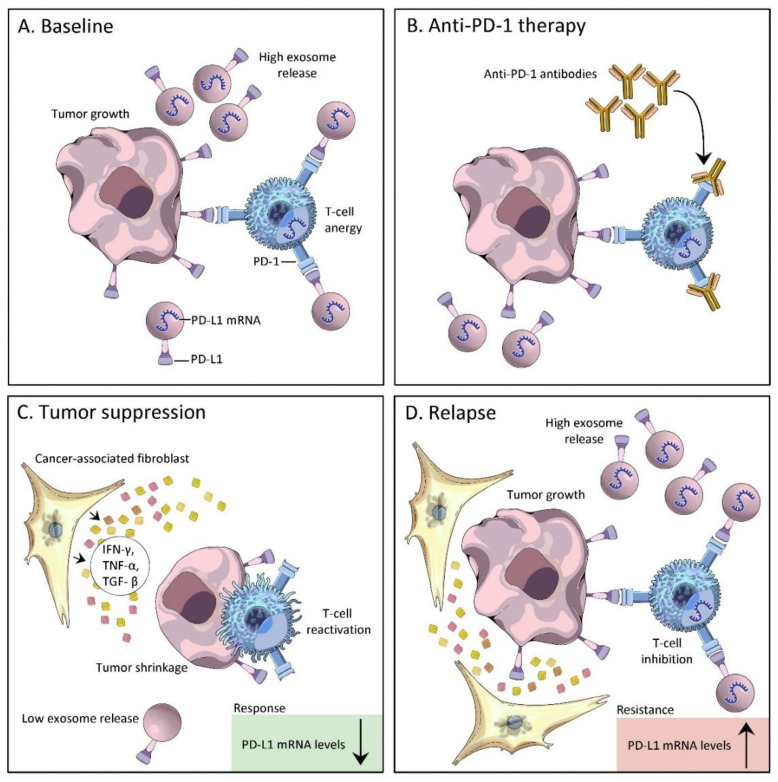
Exosomal PD-L1 correlates with tumor response and resistance to anti-PD1 therapy: (**A**) Tumor cell-derived extracellular vesicles cause immune suppression by the direct engagement of PD-1 on T cells (**B**) PD-L1/PD-1 interaction is blocked by the presence of anti-PD-1 monoclonal antibody (**C**) Tumor suppression: PD-L1 expression levels in exosomes are inversely related to the tumor’s response to immunotherapy. PD-L1 mRNA levels significantly declined from the start of treatment in patients with complete and partial responses to anti-PD-1therapy, characterized by low exosome release, T cell reactivation, and tumor shrinkage. (**D**) Tumor relapse: PD-L1 expression levels in exosomes are directly related with tumor resistance to immunotherapy. PD-L1 mRNA significantly increased in patients with a tumor relapse, characterized by increased exosome release, T-cell inhibition, and tumor growth. Downwards arrow—decreased, Upwards arrow—increased.

The most investigated subpopulation of EVs are small EVs, also known as exosomes (30-100 nm diameter) [52]. Tumor cells release extracellular vesicles in the form of exosomes carrying PD-L1 on their surface. The membrane topology of exosomal PD-L1 is similar to the PD-L1 cell surface. Exosomal PD-L1 binds to the receptor PD-1 on the surface of T lymphocytes, restricting their activity and consequently decreasing the efficiency of anti-PD-L1 therapy [45]. Recent studies have demonstrated that extracellular vesicles isolated from the blood samples of cancer patients have significantly higher levels of PD-L1 as compared to those of healthy donors [53,54,55]. Moreover, the levels of PD-L1 on circulating extracellular vesicles in cancer patients receiving anti-PD-1 therapy vary considerably between the clinical responder and non-responder groups, as observed in a study on melanoma patients [56]. Patients who failed to respond to the anti-PD1 therapy had higher pre-treatment levels of circulating exosomal PD-L1, indicating the exhaustion of T cells and their inability to be renewed by anti-PD1 treatment. Increased levels of PD-L1 on circulating exosomes was exhibited by clinical responders within 3 to 6 weeks of therapy, which suggested a positive correlation of PD-L1 levels to T cell rejuvenation and successful induction of anti-tumor immunity by anti-PD1 therapy [45]. The levels of circulating exosomal PD-L1 before and during anti-PD1 therapy can thus be illustrative of distinct states of anti-tumor immunity. Since the expression levels of PD-L1 in tumor-derived exosomes are significantly associated with immune response and cancer progression, exosomal PD-L1 could be harnessed as a biomarker for contemplating cancer therapy responses (Figure 4).

Studies have suggested the correlation between the total protein content in small EVs and survival rate in cancer patients. Increased amounts of proteins associated with circulating small EVs have been found in lung and breast cancer patients with brain metastases [57]. PD-L1 secreted in plasma small EVs can be used to identify the immunotherapy response in melanoma patients [58]. MicroRNAs (miRNAs) are one of the key signaling molecules of small EVs. These are small non-coding RNAs, 21–25 nucleotides long, and can be packaged well into small EVs and regulate multiple target genes [59]. miRNAs in cancer cell-derived small EVs have been shown to assist in tumor progression and immune evasion. The molecular analysis of PD-L1 expression in small EVs from colorectal cancer (CRC) patients has identified the synergistic role of miR-21-5p and miR-200a in the regulation of PD-L1 expression in tumor-associated macrophages (TAMs) [60]. miR-21-5p is associated with the regulation of cell proliferation, migration, and apoptosis [61,62,63,64]. miR-200a belongs to the miR-200 family and has an oncogenic role in certain types of human cancer such as gastric cancer, esophageal cancer, hepatocellular carcinoma, breast cancer, etc. [65,66,67,68,69,70]. Together they promote TAM-mediated inhibition of CD8+ T lymphocytes, thereby contributing to immune escape and CRC progression. Small EVs thus hold great prognostic and diagnostic potential [60]. 

There is a heterogeneous population of extracellular vesicles larger than exosomes, ranging from a hundred nanometers to a few microns, and mostly derived from the plasma membrane [71,72]. Large oncosomes (LO) are extracellular vesicles in the size range of 1–10 μm which are shed particularly by cancer cells, and have been described in prostate cancer [73], breast cancer [74], pancreatic cancer [75], colon cancer [76], melanoma [77], etc. LO have a different composition than the vesicles shed from normal cells and/or tumor microenvironment [78], and can contain a larger number of tumor-derived molecules owing to their size [79]. The ability to encase diverse molecular cargo makes LO advantageous as potential diagnostic/prognostic biomarkers for cancer (Table 3).

### 4.2. Bacterial Extracellular Vesicles (bEVs)

**Figure 5:**
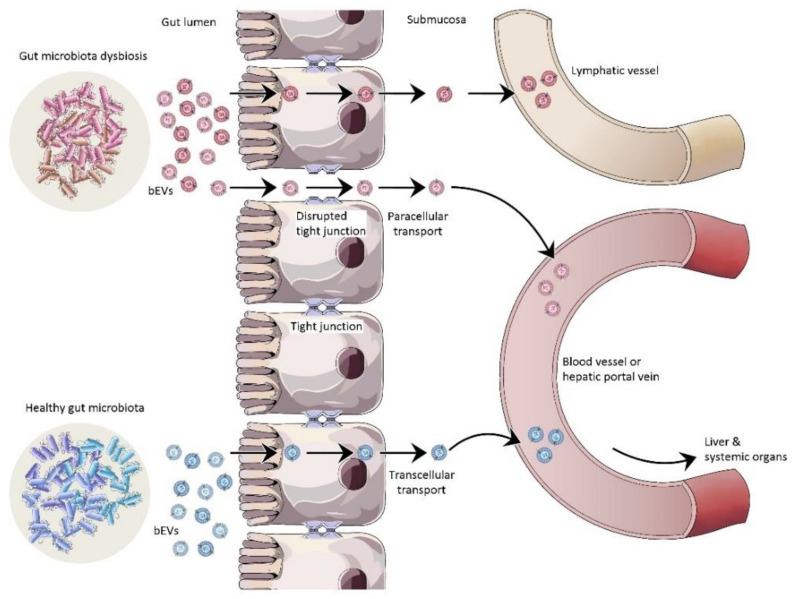
In healthy conditions, luminal epithelium is intact and fewer bEVs can pass transcellularly into the systemic circulation. In microbial dysbiosis, intestinal barrier dysfunction facilitates rapid transport of bEVs into the systemic circulation, induction of immune activation, and intervention in carcinogenesis/tumor progression.

bEVs are nano-sized particles released by Gram-negative and certain Gram-positive bacteria that are bound by a lipid membrane and consist of bacteria-derived components. bEVs are involved in bacteria–bacteria and bacteria–host interactions. Recent studies suggest that bEVs could impact oncogenesis and tumor progression [88,89]. Microbial dysbiosis has been established as a crucial determinant in the regulation of oncogenesis and tumor progression, especially in GI-tract-related malignancies such as gastric, colorectal, liver, and pancreatic cancer, etc., as well as in the response to therapy [11,12,13]. The mechanism underlying microbial effect on carcinogenesis and tumor progression remains largely unexplored. Since bEVs have been detected in blood circulation [14], it can be hypothesized that the gut microbiome intervenes in carcinogenesis with the aid of systemically circulating bEVs that immunomodulate recipient cells in distant organs. Tight junctions in the luminal epithelium might be disrupted as a consequence of microbial dysbiosis allowing bEVs to passively pass into the submucosa and eventually into the circulatory system and lymphatics for systemic dissemination [90]. In normal conditions where gut luminal epithelium is intact, a smaller number of bEVs can escape to the underlying submucosa and subsequently to the systemic circulation by active transcellular transport [14] (Figure 5).

#### 4.2.1. bEVs as Diagnostic Markers

Increasing evidence indicates the correlation between disease-associated microbiome changes and bEV levels in the biofluids and their composition. Serum-derived bEVs have emerged as a promising tool for the diagnosis of various diseases such as cancer [91]. Similarly, bEVs can also be excreted via the urinary tract and have been found to be constantly altered in the urine in a diseased state, indicative of associated microbiome changes [92,93]. The presence of specific bEVs in biofluids can be linked to a specific state of a cancer disease [91], making bEVs enticing biomarkers for clinical diagnosis [76,91]. Metagenomic and metabolomic analyses of vesicles isolated from feces of colorectal cancer patients have demonstrated the interrelationship between microbial dysbiosis and metabolic alternations within the vesicle population, indicating that dynamic alterations in the metabolic information carried by the gut-derived bEVs infer the health state of the host [94].

#### 4.2.2. bEVs as Cancer Immunotherapy Agents

Gram-negative bacteria cast off prokaryotic vesicles, known as outer membrane vesicles (OMVs), that have gained increased attention as the next generation vaccine carrier owing to their high immunogenicity, mutable genome, ability to target lymph nodes, and ability to carry heterologous antigens [95]. In the early 1890s, Dr. William Coley injected the solution of attenuated bacteria in cancer patients as a treatment, which was reported as the first incidence of the application of bacteria-associated substances to treat cancer [96]. Kim et al. (2017) showed that bEVs accumulate in tumor tissues in mice and activate anti-tumor immune response via the IFN-γ signaling pathway [97]. This was the first reported study proposing use of bEVs as cancer immunotherapeutic agents. 

Regardless of the various advantages of OMVs as potent cancer adjuvants, they can trigger severe innate immune responses in vivo such as sepsis, cardiomyopathy, and pulmonary diseases through their pro-inflammatory components, such as lipopolysaccharide (LPS) and other virulence factors [98,99,100]. To counteract these adverse effects and enhance the tumor immunotherapeutic potential, combination therapy incorporating modified bEVs has become a prerequisite for full eradication of the tumor and prevention of tumor recurrence and metastasis. Cancer therapy research has been advancing in accordance with this approach. For instance, Li et al. (2020) developed *E. coli* OMVs coupled with the ectodomain of the immune checkpoint PD-1 on their surface, which augmented the aggregation of OMVs at the tumor site and induced a PD-L1 blockade effect. These engineered OMVs enhanced the anti-tumor immune responses by ~1.5-fold as compared to the treatment with natural OMVs [101]. Attempts to produce detoxified OMV-like vesicles called synthetic bacterial vesicles (SyBV) by specific biochemical processes, which can be used in combination with tumor derived extracellular vesicles (tEV) as immunotherapy, have been in progress. The immunotherapeutic potential of such combinations of SyBV and tEV to induce humoral and cellular immunity, leading to effective anti-tumor activity, has been tested in vivo in melanoma and colon cancer mice models [102]. Another combination of very small size particles (VSSP) and *Neisseria meningitidis*-derived outer membrane vesicles is being developed as a nanoparticle-based immunomodulator in ovarian cancer patients [103]. 

In a recent report, Gram-negative bEVs derived from a genetically modified, endotoxin-free *Escherichia coli* (*E. coli*) strain showed selective tropism for tumor tissues when administered systematically, and induced lasting anti-tumor immune responses through the production of cytokines CXCL10 and interferon-γ, with no adverse reactions [97]. Gram-positive bEVs derived from *Lactobacillus acidophilus* and *Staphylococcus aureus* also showed similar anti-tumor effects [97]. All these studies emphasize blending traditional treatment strategies with natural or modified bEVs to boost the efficacy of current cancer treatments [104,105]. However, studies pertaining to the applications of bEVs in cancer research are still in their infancy and need thorough scrutinization.

## 5. Conclusions

Compelling evidence is present that suggests human gut microbiota influences therapeutic responses to cancer immunotherapy by manipulating the host immune mechanism [12]. Positive attempts have been made to use the beneficial gut microbiota of patients who have recovered from cancer as a potential therapeutic method to improve the immune capability of patients not responding to immunotherapy via fecal microbial transplant [12,22,42]. From a therapeutic point of view, it would be more desirable to administer bEVs from the responders to non-responders as an alternative to FMT.

Immune checkpoint blockade therapy has provided outstanding clinical effects for cancer patients to effectively prolong their overall survival period. The effect of this therapy has been found to be heterogenous and successful only in a minority of populations [37]. One of the factors influencing the efficacy of the therapy has been identified as the gut microbial population of the patients [12,13]. Several studies have been conducted to identify the microorganisms that positively influence the anti-PD-1/PDL-1 treatment outcomes to provide maximum benefits to patients in a cost-effective way [37]. In a similar line, bEVs that may have a positive influence in the treatment outcome can be identified and used as biomarkers for tracking the tumor progression and therapy response.

**Figure 6:**
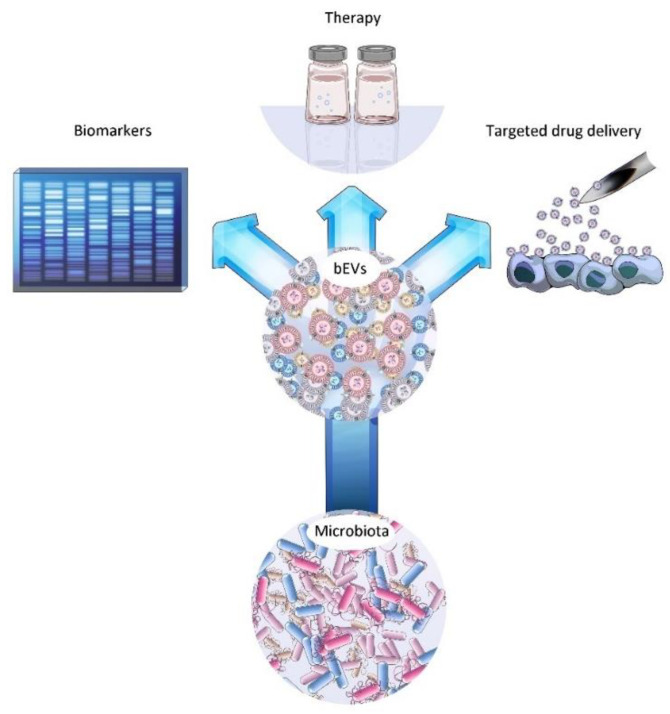
Potential applications of bacterial extracellular vesicles.

The nanosized and non-replicative status of bEVs, their ability to be bioengineered to produce desired effect [106,107], their accumulation near the tumor sites, their capacity to induce anti-tumor immune responses [15,108], and their ability to carry the desired payloads [109,110] provide lucrative incentives for the further study of bEVs in order to fill the current knowledge gap as well as to develop optimized novel cancer therapy modules (Figure 6).

## Figures and Tables

**Table 1:** Studies depicting interaction of microbiota in anti-PD-1/PD-L1 therapy.

Studied by	Study Group	Disease Studied	Treatment Used	Microorganism Involved	Clinical Response to Therapy
Peng et al. [25]	Human	Gastrointestinal cancer	Anti–PD-1/PD-L1	*Bacteroidetes*, *Firmicutes*	High in responders
Routy et al. [13]	Human	Non-small cell lung cancer (NSCLC) and renal cell carcinoma (RCC)	Anti–PD-1	*Akkermansia muciniphila*	High in responders
Matson et al. [12]	Human	Metastatic melanoma	Anti–PD-1	*Bifidobacterium longum*, *Collinsella aerofaciens*, *Enterococcus faecium*	High in responders
Frankel et al. [27]	Human	Metastatic melanoma	Anti-PD-1 and Anti-CTLA4	*Bacteroides caccae*, *Dorea formicogenerans*	High in responders
Gopalkrishnan et al. [22]	Human	Metastatic melanoma	Anti–PD-1	*Ruminococcaceae* family	High in responders
Chu et al. [28]	Human	Lung cancer	Anti–PD-1	*Fusobacterium*	Produced resistance to Anti–PD-1 therapy
Zheng et al. [29]	Human	Hepatocellular carcinoma (HCC)	Anti–PD-1	*Akkermansia muciniphila* & *Ruminococcaceae* spp.	High in responders
Sivan et al. [19]	Mice	Melanoma	Anti-PD-L1	*Bifidobacterium*	Promotes anti-tumor immunity

**Table 2:** Studies involving fecal microbiota transplant (FMT) in anti-PD-1 therapy.

Study Done by	Study Model	Disease Studied	Clinical Response in Patients Receiving FMT
Gopalakrishnan et al. [22]	Germ free mice	Metastatic melanoma	Improved responses to anti–PD-L1 therapy; significant reduction in tumor size
Matson et al. [12]	Germ free mice	Metastatic melanoma	Showed slow tumor growth
Routy et al. [13]	Germ free mice	Non-small cell lung cancer (NSCLC)	Delay in tumor growth
Baruch et al. [41]	Human	Metastatic melanoma	Out of 10 PD-1–refractory metastatic melanoma patients, 3 patients showed improved response to anti-PD-1 therapy
Davar et al. [42]	Human	Metastatic melanoma	Out of 15 PD-1–refractory metastatic melanoma patients, 6 patients showed improved response to anti-PD-1 therapy

**Table 3:** Cargo composition of Large Oncosomes.

Molecules		Functions	References
Nucleic acids	*MYC*, *AKT1*, *PTK2*, *KLF10*, *PTEN*	Genes encoded by chromosomal DNA, their copy number variations favor cancer cell progression	[80,81]
	miR-1227	Increases migration of cancer-associated fibroblasts (CAFs) when overexpressed	
	GAPDH, GPI, LDHB, HSPA5, MDH, GOT, GLS	Metabolic enzymes	[82]
	V-ATPase subunit V1G1	Promote tumor progression by delivering oncogenic signals and reprogramming the tumor microenvironment	
Proteins	Urokinase-type plasminogen activator receptor (uPAR)	Promote cancer progression when released by the aggressive counterpart	[73,83]
	Eukaryotic elongation factor 1 gamma (eEF1γ)		
	Serine-threonine protein kinase AKT1		
	Caveolin-1, CK18, MMP 2, MMP9	Scaffolding protein/ cytoskeleton components and gelatinase activity	[73,84,85]
	Small GTP-binding protein ARF6	Coordinates the release of plasma membrane-derived microvesicles containing protease from tumor cells into the surrounding environment	[86,87]
	αV-integrin	Imparts the adhesive and invasive properties of aggressive cancer cell line to the less aggressive equivalent	[73]

## Data Availability

Not applicable.

## Supplementary Materials

The following supporting information can be downloaded at: https://www.mdpi.com/article/10.3390/cancers14205121/s1, Table S1: Scopus and PubMed query search terms and results.
