# Supplementary material for: Microbiota and Extracellular Vesicles in Anti-PD-1/PD-L1 Therapy
Source: Cancers (Basel). 2022 Oct 19;14(20):5121. doi: 10.3390/cancers14205121 (PMC9600290; doi:10.3390/cancers14205121)
Supplement: Supplementary file 1 [file cancers-14-05121-s001.zip › cancers-1899008-supplementary.pdf]

Supplementary Table S1. Scopus and PubMed query search terms and results.

**Scopus Query search on 09-06-2022**	**Items found/ included**
(TITLE-ABS-KEY (exosome OR "extracellular vesicle*" OR "outer membrane vesicle*" OR "micro vesicle*") OR TITLE-ABS-KEY ("microbio*" OR "bacteri*") AND TITLE-ABS-KEY ("anti-pd*")) AND (LIMIT-TO (PUBSTAGE , "final")) AND (LIMIT-TO (LANGUAGE , "english"))	377
Language filter	English
**PubMed Query search on 09-06-2022**	
((extracellular vesicle[MeSH Terms]) OR (microbio*[MeSH Terms] OR bacteria*[MeSH Terms])) AND (anti-PD*[Text]) Filters: English	355
Language filter	English
Duplicates	190
Total screened after duplicates removal	522
Total articles after exclusion with reasons (reason: Not fitting the content designed for the review)	97

Registration information: The manuscript has been registered to PROSPERO on 13.06.22 with the ID CRD42022336985.
